# Nordalbergin Synergizes with Novel *β*-Lactam Antibiotics against MRSA Infection

**DOI:** 10.3390/ijms25147704

**Published:** 2024-07-14

**Authors:** Haiting Wang, Sangyu Hu, Yuzhu Pei, Hongxiang Sun

**Affiliations:** 1 College of Animal Sciences, Zhejiang University, Hangzhou 310058, China; whting1982@163.com; 2 State Key Laboratory for Diagnosis and Treatment of Severe Zoonotic Infectious Diseases, Institute of Zoonosis, College of Veterinary Medicine, Jilin University, Changchun 130062, China; hsangy@163.com (S.H.); peiyuzhu2024@163.com (Y.P.); 3 Key Laboratory for Zoonosis Research of the Ministry of Education, Institute of Zoonosis, College of Veterinary Medicine, Jilin University, Changchun 130062, China

**Keywords:** nordalbergin, *β*-lactamases, *β*-lactam antibiotics, antibiotic synergist, MRSA

## Abstract

The synergetic strategy has created tremendous advantages in drug-resistance bacterial infection treatment, whereas challenges related to novel compound discovery and identifying drug-binding targets still remain. The mechanisms of antimicrobial resistance involving *β*-lactamase catalysis and the degradation of *β*-lactam antibiotics are being revealed, with relevant therapies promising to improve the efficacy of existing major classes of antibiotics in the foreseeable future. In this study, it is demonstrated that nordalbergin, a coumarin isolated from the wood bark of *Dalbergia sissoo*, efficiently potentiated the activities of *β*-lactam antibiotics against methicillin-resistant *Staphylococcus aureus* (MRSA) by suppressing *β*-lactamase performance and improving the bacterial biofilm susceptibility to antibiotics. Nordalbergin was found to destabilize the cell membrane and promote its permeabilization. Moreover, nordalbergin efficiently improved the therapeutic efficacy of amoxicillin against MRSA pneumonia in mice, as supported by the lower bacterial load, attenuated pathological damage, and decreased inflammation level. These results demonstrate that nordalbergin might be a promising synergist of amoxicillin against MRSA infections. This study provided a new approach for developing potentiators for *β*-lactam antibiotics against MRSA infections.

## 1. Introduction

Methicillin-resistant *Staphylococcus aureus* (MRSA) was first discovered in 1960, and is resistant to methicillin and other *β*-lactam antibiotics; it is prevalent in many healthcare facilities worldwide and has become a global priority for infection control efforts [1,2]. Despite the growing resistance, *β*-lactam antibiotics remain among one of the most widely used antimicrobial agents in aquaculture, agricultural, and clinical settings. The discovery of the first *β*-lactam antibiotic (penicillin G) is an important milestone in modern chemotherapy. And the increasing number of other *β*-lactam antibiotics have been introduced into clinical therapy to revolutionize the routine therapies for bacterial infections [3,4,5]. Nowadays, novel fourth- and fifth-generation cephalosporins such as cefpirome, ceftaroline, and ceftobiprole have been developed [6]. However, the emergence and prevalence of multidrug-resistant (MDR) bacteria posed an increasing and urgent requirement to address this crisis.

To date, compared to the development of new antimicrobial drugs, combination therapy with existing antibiotics and adjuvant enhancers has attracted increasing interest due to unique properties such as low costs and potential applications in solving MRSA infections [7]. One of the best examples is the synergistic combination of *β*-lactam antibiotics with *β*-lactamase inhibitors such as clavulanic acid and avibactam, which greatly highlighted the therapeutic potential of discovery the new *β*-lactamase inhibitors as a feasible anti-MDR bacterial infection strategy [8]. In addition, it has been reported that triterpenoid saponin, flavonoids, and coumarins could increase the sensitivity of MRSA to *β*-lactam or aminoglycoside antibiotics [9,10,11]. Collectively, these findings solidified its efficacious and promising feasibility of combined therapy in controlling the survival of MRSA.

**Figure 1:**
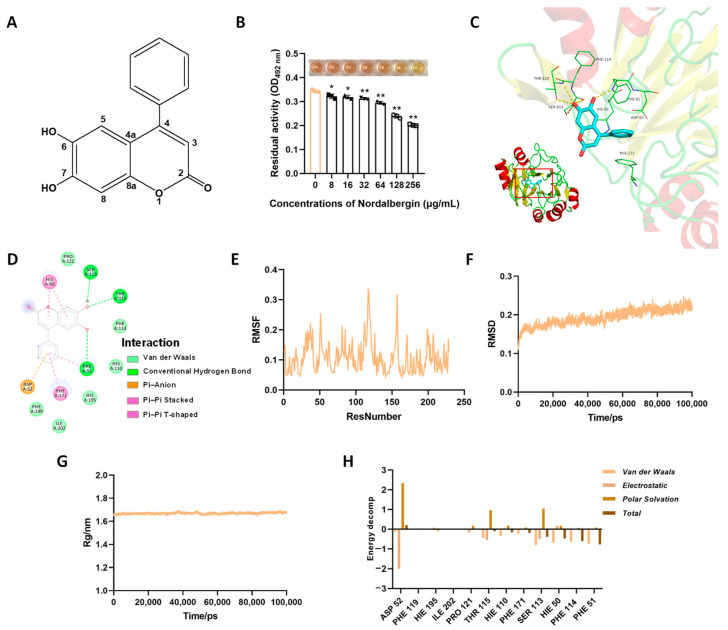
Nordalbergin suppressed *β*-lactamase activity by interacting with the active residues. (**A**) Chemical structure of nordalbergin. (**B**) The effect of nordalbergin on *β*-lactamase activity by an enzyme inhibitory assay. The data are expressed as means ± SD (*n* = 3). * *p* < 0.05 and ** *p* < 0.01 vs. 0 μg/mL. (**C**,**D**) The binding mode between nordalbergin ligand and *β*-lactamase by molecular dynamics simulation. (**E**–**G**) The stability of *β*-lactamase/nordalbergin complex by root mean square fluctuation (RMSF, **E**), root mean square deviation (RMSD, **F**), and radius of gyration (Rg, **G**). (**H**) The residue energy decomposition analysis of the key amino acid residues involved in the binding process of *β*-lactamase and nordalbergin.

Nordalbergin (Figure 1A) is a 4-phenyl coumarin compound isolated from the wood bark of *Dalbergia sissoo* Roxb. (Papilionaceae). So far, nordalbergin has not been well investigated except that it has been reported to induce the terminal differentiation of human promyelocytic leukemia HL-60 cells toward mature monocytes/macrophages [12] as well as possess anti-inflammatory and antioxidant activities in LPS-stimulated microglial cells [13]. In this study, the synergistic antibacterial activity of nordalbergin and *β*-lactam antibiotics against MRSA was investigated through a checkerboard assay, combined disk test, and time-dependent killing curves. Nordalbergin was found to synergistically counteract MRSA with *β*-lactam antibiotics. Meanwhile, the inhibitory effects and the underlying mechanisms of action were evaluated by the nitrocefin assay and molecular dynamics simulation study. Furthermore, the improved therapeutic efficacy of nordalbergin on amoxicillin against MRSA pneumonia was evaluated in mice. This study presented a new *β*-lactam antibiotic potentiator to synergistically combat the MRSA infection.

## 2. Results

### 2.1. Nordalbergin Inhibited the Activity of β-Lactamase

Increasing antimicrobial resistance revitalized *β*-lactamase inhibitor research. The effect of nordalbergin on *β*-lactamase activity was determined using *β*-lactamase protein by an enzyme inhibitory assay [14]. As shown in Figure 1B, nordalbergin significantly inhibited the *β*-lactamase activity, as supported by a proportional decrease in absorbance.

Molecular simulation revealed the potential interactions between *β*-lactamase and nordalbergin (Figure 1C). As shown in Figure 1D, residues HIS50, PHE171, and ASP52 are involved in Pi-Pi stacked, Pi-Pi T-shaped, and Pi-Anion interactions while the important residues PRO121, PHE114, HIS110, HIS195, ILE202, and PHE199 are involved in van der Waals interactions. Further, root mean square fluctuation (RMSF), root mean square deviation (RMSD), and radius of gyration (Rg) were assessed to determine the stability of the *β*-lactamase/nordalbergin complex. The RMSF values reflected the fluctuations in amino acids in this complex (Figure 1E). The conformational stability within this system is shown by the changes in the overall spectrum of RMSD during the simulation (Figure 1F). The Rg showed the structural compactness and equilibrium of the complex (Figure 1G). Importantly, the energy decomposition of key amino acid residues revealed the attachment and strength of the interactions as required for the inhibition efficacy of nordalbergin against *β*-lactamase activity (Figure 1H). These results together indicated the potential interactions of nordalbergin and *β*-lactamase.

### 2.2. Synergistic Efficacy of Nordalbergin and β-Lactam Antibiotics against MRSA

**Figure 2:**
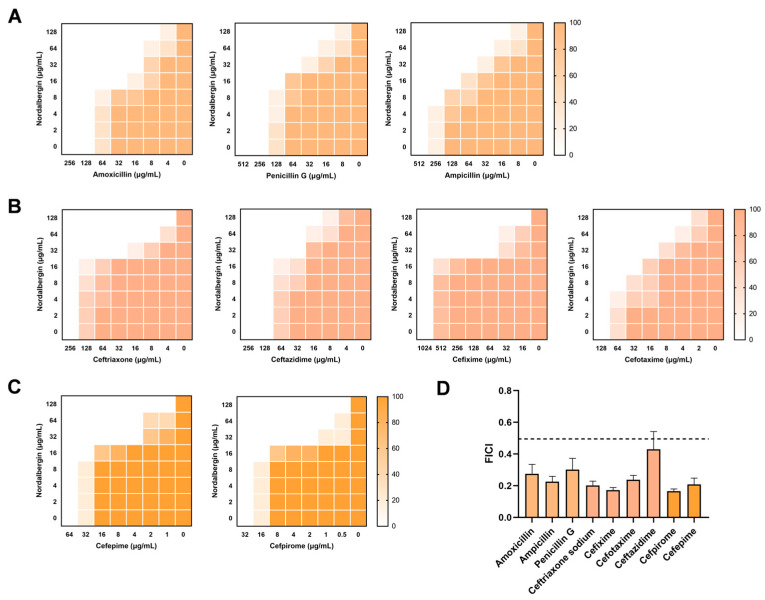
Synergistic antimicrobial activities of nordalbergin and *β*-lactam antibiotics against MRSA. (**A**–**C**) The combination of nordalbergin and penicillin antibiotics (amoxicillin, ampicillin, and penicillin G, **A**), third-generation cephalosporins (ceftriaxone, cefixime, cefotaxime, and ceftazidime, **B**), and fourth-extended-spectrum cephalosporins (cefpirome and cefepime, **C**) against MRSA. The subfigures (**A**–**C**) shown are representative of five independent experiments. (**D**) The fractional inhibitory concentration index (FICI) values of nordalbergin (32 μg/mL) in combination with the above antibiotics.

To assess the synergistic efficacy of nordalbergin and *β*-lactam antibiotics, the modified checkerboard method was employed. As expected, in the presence of nordalbergin (≥32 μg/mL), all MIC values against MRSA for *β*-lactam antibiotics including amoxicillin, ampicillin, penicillin G (Figure 2A), and third-generation cephalosporins (ceftriaxone, cefixime, cefotaxime, and ceftazidime, Figure 2B), and fourth-extended-spectrum cephalosporins (cefpirome and cefepime, Figure 2C) were decreased by ≥4-fold. Their fractional inhibitory concentration index (FICI) values were all less than 0.5, indicating an effective synergistic effect between nordalbergin and *β*-lactam antibiotics (Figure 2D). 

**Figure 3:**
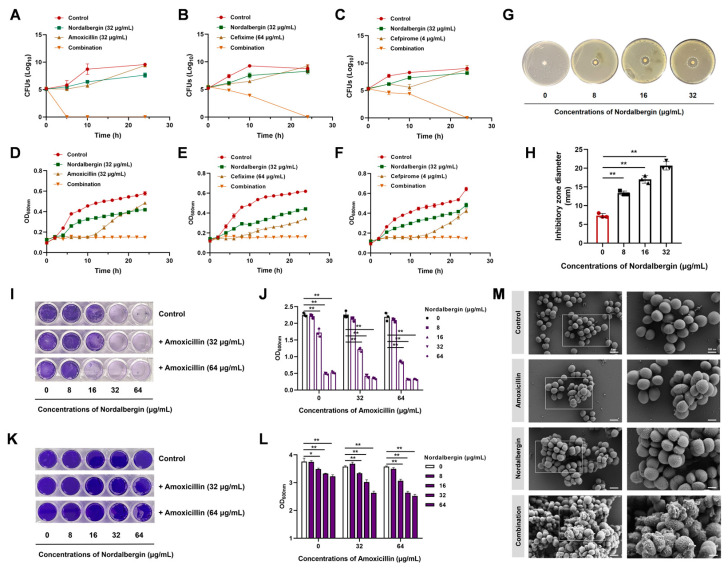
Nordalbergin boosted the antimicrobial activity and biofilm inhibition of *β*-lactam antibiotics. (**A**–**F**) Time-dependent killing curves (**A**–**C**) and cell viability (**D**–**F**) of MRSA of the combination of nordalbergin and *β*-lactam antibiotics (amoxicillin, cefixime, and cefpirome). (**G**,**H**) The combined disk assay of nordalbergin in combination with amoxicillin against MRSA. (**I**–**L**) Effect of nordalbergin combined with amoxicillin on the bacterial biofilm formation (**I**,**J**) and eradication of mature biofilms (**K**,**L**) by crystal violet staining. (**M**) Morphological changes of MRSA treated with amoxicillin/nordalbergin alone or their combinations under SEM. The figure shown is representative of three independent experiments. The data are expressed as means ± SD for three independent experiments. * *p* < 0.05 and ** *p* < 0.01.

As shown in Figure 3A–C, nordalbergin combined with the representative *β*-lactam antibiotics (amoxicillin, cefixime, or cefpirome) exhibited a competently bactericidal activity against MRSA compared to the antibiotics (amoxicillin, cefixime, or cefpirome) or nordalbergin alone, as evidenced by a complete elimination within 24 h. Consistently, nordalbergin alone exerted negligible impact on the growth of MRSA, whereas bacterial growth was significantly suppressed by its combination with the above *β*-lactam antibiotics (Figure 3D–F). The disk diffusion assay further indicated that nordalbergin increased antimicrobial susceptibility to amoxicillin in a dose-dependent manner (Figure 3G,H).

In light of the importance of biofilm formation in the pathogenicity of drug-resistant bacteria [15,16], the anti-biofilm effects of nordalbergin were investigated. As shown in Figure 3I,J, the combination of nordalbergin and amoxicillin effectively eradicated the biofilm of MRSA and exerted a potential synergistic efficacy. Furthermore, nordalbergin combined with amoxicillin displayed a potent inhibitory effect on the maturation of microbial biofilms (Figure 3K,L). These findings highlighted the antimicrobial and antibiofilm dual function of nordalbergin as an amoxicillin adjuvant to tackle biofilm-related multi-drug-resistant bacteria. SEM observations further visualized the morphological membrane damage induced by the combination of nordalbergin and amoxicillin in comparison with nordalbergin/amoxicillin alone (Figure 3M).

### 2.3. Nordalbergin Synergized with β-Lactam Antibiotics against MRSA by Enhancing Cell Membrane Permeability

**Figure 4:**
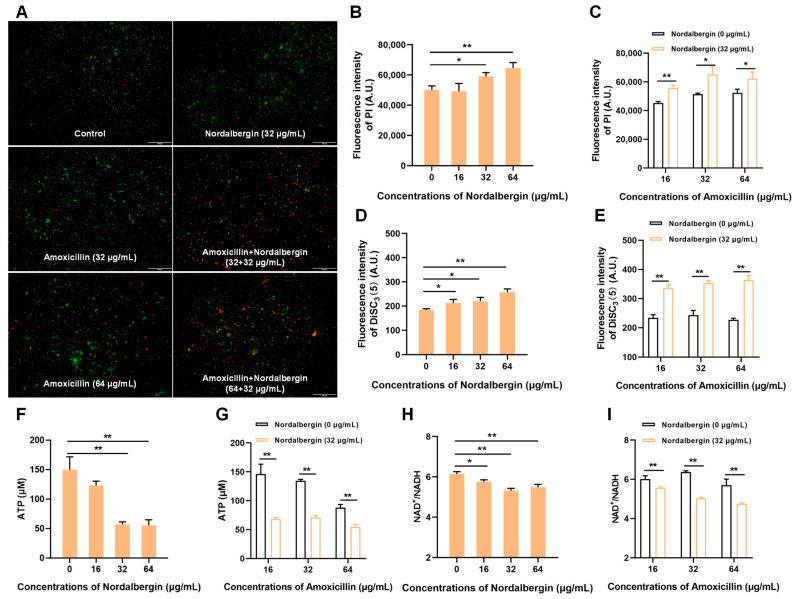
Nordalbergin impaired cell membrane function. (**A**) The bacterial viability using live/dead bacteria staining (live cells were labeled green and dead cells were dyed red). The figure shown is representative of three independent experiments. (**B**,**C**) Cell membrane permeability of MRSA using propidium iodide (PI) staining. (**D**,**E**) Membrane potentials of MRSA using DiSC_3_(5) probe. (**F**,**G**) The intracellular ATP levels of MRSA. (**H**,**I**) NAD^+^/NADH levels of MRSA. The data are expressed as means ± SD (*n* = 3). * *p* < 0.05 and ** *p* < 0.01.

The fluidity and permeability of cell membranes are crucial for normal cellular function. As shown in Figure 4A, nordalbergin combined with amoxicillin significantly enhanced the proportion of PI-stained dead cells (red fluorescence) compared with nordalbergin/amoxicillin alone. Moreover, the membrane permeability was significantly enhanced by the combination of nordalbergin and amoxicillin, as evidenced by the increased fluorescence intensity of PI (Figure 4B,C). Both nordalbergin alone and combined with amoxicillin markedly dissipated the membrane potentials (Figure 4D,E). These results together suggested that nordalbergin impaired cell membrane function resulting in potentiated antibacterial activity. Meanwhile, given that the destruction of membrane potential would affect adenosine triphosphate (ATP) synthesis, the intracellular ATP levels were assessed and the results were shown in Figure 4F,G. Both nordalbergin alone and combined with amoxicillin significantly decreased the intracellular ATP levels. Furthermore, the tricarboxylic acid (TCA) cycle metabolism level in bacteria exposed to diverse concentrations of nordalbergin or combined with amoxicillin were also analyzed. Both nordalbergin alone and combined with amoxicillin significantly enhanced the TCA cycle compared to monotherapy (Figure 4H,I). Collectively, these findings suggested that nordalbergin exerts potent synergistic antibacterial activity with *β*-lactam antibiotics through inducing membrane destabilization and the consequent permeabilization.

### 2.4. Nordalbergin Improved Therapeutic Efficacy of Amoxicillin against MRSA Pneumonia in Mice

**Figure 5:**
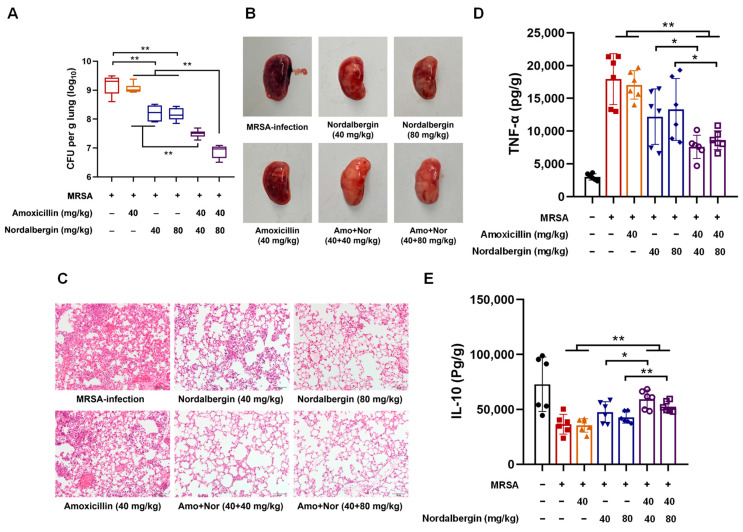
Nordalbergin enhanced the therapeutic efficacy of amoxicillin against MRSA pneumonia in mice. (**A**) The bacterial load in the lung tissues of MRSA pneumonia mice. (**B**) Lung anatomy of MRSA pneumonia mice. (**C**) The histopathological changes in lung tissues in MRSA pneumonia mice by H&E staining. The light photomicrographs shown were representative of lung tissue sections from six mice per group. Scale bar = 50 μm. (**D**,**E**) Levels of TNF-α (**D**) and IL-10 (**E**) in lung tissues of MRSA pneumonia mice by ELISA. The data are expressed as means ± SD (*n* = 6). * *p* < 0.05 and ** *p* < 0.01.

The mouse MRSA pneumonia model was established to evaluate the therapeutic efficacy of the combination of amoxicillin and nordalbergin. As shown in Figure 5A, the combination of amoxicillin and nordalbergin observably decreased bacterial loads in lung tissues compared with amoxicillin or nordalbergin monotherapy. Similarly, pulmonary lesions in MRSA-infected mice were significantly alleviated by the combination of amoxicillin and nordalbergin, as evidenced by the mitigant congestion and hemorrhage, as well as reduced inflammatory cells infiltrating (Figure 5B,C). Furthermore, the effects of the combination treatments on inflammatory responses in lung tissues were also evaluated using ELISA. Compared to the model control and amoxicillin/nordalbergin-alone groups, the level of pro-inflammatory cytokine TNF-α was markedly reduced (Figure 5D), whereas that of anti-inflammatory cytokine IL-10 was significantly increased (Figure 5E) in lung tissues by the combination treatment of amoxicillin and nordalbergin. These data demonstrated that nordalbergin potently enhances the therapeutic efficacy of amoxicillin against MRSA pneumonia in mice.

## 3. Discussion

The *β*-lactam antibiotics including penicillin, cephalosporins, and carbapenems, one of the most widely used and effective classes of antibacterial agents, provided a strategy to tackle bacterial infections by the suppression of bacterial wall biogenesis [17]. *β*-lactamases mediated enzymatic catalyzation and degradation of the *β*-lactam ring of *β*-lactam antibiotics resulting in a considerable obstacle to the rational use of antibiotics and the emergence and prevalence of drug-resistant bacteria. In the current study, to overcome the resistance by *β*-lactamase in *S. aureus*, especially MRSA, a bioactive coumarin compound nordalbergin was found to significantly inhibit the *β*-lactamase activity, and further work synergically against MRSA with diverse generations of *β*-lactam antibiotics including penicillin, and third- and fourth-generation cephalosporins. 

Notably, accumulating evidence suggested that natural and synthetic coumarins were developed as therapeutic agents with a plethora of pharmacological effects such as antioxidant, antimicrobial, antiangiogenic, and anticancer activities; in addition, a series of novel and commercially available drugs comprising coumarin as cap groups exhibited favorable and efficacious therapeutic potential [18,19,20]. The antineoplastic and anti-neuroinflammatory effects of nordalbergin have been reported; however, its other multifaceted benefits are still not fully elucidated. Herein, nordalbergin was evaluated for synergistic antibacterial activity with *β*-lactam antibiotics in vitro and in vivo; its molecular mechanism was also explored.

Intriguingly, compared with the model control group, nordalbergin alone exhibited a high therapeutic potential. However, the in vitro antimicrobial test revealed that the MIC value of nordalbergin against MRSA was >128 μg/mL, suggesting that its anti-infective action might be explained by the reduced virulence of the pathogens, rather than attributed directly to antibacterial properties. *S. aureus* utilizes a plethora of complex virulence factors including toxins and proteases to enhance bacterial survivability and thrive in divergent physiological contexts [21,22]. Nowadays, targeting the key virulence factors without inducing direct lethality has attracted increasing interest as a promising anti-infection strategy. The in vivo data enlighteningly suggested that the therapeutic potential of nordalbergin alone might be attributed to diminished pathogenicity by targeting bacterial virulence, thus providing an intriguing prospect for its underlying mechanisms. In addition, it was reported that nordalbergin exhibited anti-inflammatory and anti-oxidative activities through inhibiting the MAPK signaling pathway, NLRP3 inflammasome activation, and ROS production in LPS-stimulated BV2 microglia [13], which might also be one of the reasons for improving the therapeutic efficacy of amoxicillin against MRSA pneumonia in mice. Notably, the synergistic effect of nordalbergin due to its excellent anti-*β*-lactamase performance and remarkable improvement in bacterial biofilm susceptibility to antibiotics broadened the applications in MRSA-infection diseases.

## 4. Materials and Methods

### 4.1. Bacterial Strains and Reagents

Methicillin-resistant *S. aureus* (MRSA) strain USA300 was cultivated in tryptic soy broth (TSB) at 37 °C unless otherwise stated and shaken at 160 rpm. Ceftazidime was purchased from the National Institutes for Food and Drug Control, Beijing, China; ceftriaxone, cefpirome, cefixime, cefotaxime, ampicillin, penicillin G, and amoxicillin were obtained from Dalian Meilun Biotechnology Co., Ltd., Dalian, China; cefepime was purchased from Macklin Biotechnology Co., Ltd., Shanghai, China; nordalbergin was purchased from Derick Biotechnology Co., Ltd., Chengdu, China. 

### 4.2. Enzyme Inhibition Assays

*β*-lactamase protein, responsible for the catalysis of the opening and hydrolysis of the *β*-lactam ring of *β*-lactam antibiotics, was purified as described previously [14] and incubated with diverse concentrations of nordalbergin at 37 °C for 30 min. The substrate nitrocefin (100 μg/mL) was supplemented for further incubation at 37 °C for 30 min. The inhibitory activity of *β*-lactamase was determined by monitoring the absorbance at 492 nm using a BioTek SYNERGY H1 microplate reader (Agilnet, Santa Clara, CA, USA).

### 4.3. Molecular Docking and Molecular Dynamics (MD) Simulations

The structure of *β*-lactamase (PDB ID: 7E3V) used in this study was obtained from the RCSB protein database. AutoDock Vina 1.1.2 was employed for the molecular docking of the nordalbergin/*β*-lactamase complex and MD simulations were further assessed using AMBER 18. After minimization, the nordalbergin/*β*-lactamase system was heated and equilibrated, and then a production run was executed.

### 4.4. Checkerboard Assays

Clinical and Laboratory Standards Institute (CLSI) guidelines were referenced for the checkerboard microdilution tests. Briefly, two-fold serial dilution of nordalbergin and antibiotics was performed; after 16–24 h co-incubation with bacterial suspension (5 × 10^5^ CFUs/mL) in 96-well flat-bottomed plates, the MIC was recorded as wells with the lowest concentrations of drugs with no visible growth. The FIC index was calculated according to the following formula: FIC index = (MIC of compounds in combination/MIC of compound alone) + (MIC of antibiotics in combination/MIC of antibiotics alone). The interactions were defined as synergistic when the FIC index value was ≤ 0.5, and non-synergistic when the FIC index value was between 0.5 and 4.

### 4.5. Time-Dependent Killing Curves

MRSA USA300 was cultured with indicated concentrations of nordalbergin, antibiotics (amoxicillin, cefixime, or cefpirome), and their combinations at 37 °C. And at 0 h, 5 h, 10 h, and 24 h post-infection, the cultures were removed, diluted, and spotted on TSB agar plates for counting colony-forming units.

### 4.6. Growth Curves

MRSA USA300 cultures were incubated with diverse concentrations of nordalbergin and antibiotics (amoxicillin, cefixime, and cefpirome) alone, or in combination with nordalbergin at 37 °C. The growth curves were recorded on a BioTek SYNERGY H1 microplate reader (Agilnet, Santa Clara, CA, USA) at a wavelength of 600 nm at 2 h intervals.

### 4.7. Combined Disk Tests

The logarithmic growth phase of the MRSA USA300 solution was diluted in Luria-Bertani (LB) broth to OD_600nm_ of 0.1 and spread on the soft LB agar plate, containing different concentrations of nordalbergin (0, 8, 16, and 32 μg/mL), and then the antibiotics discs were placed in the center of the plate. Then, the disks containing 20 μg amoxicillin were plated and incubated at 37 °C for 16−20 h. The diameters of the inhibition zones were recorded and calculated.

### 4.8. Biofilm Inhibition Assays

MRSA USA300 culture mixture of nordalbergin, amoxicillin, or the combination of nordalbergin plus amoxicillin were seeded in 24-well plates and incubated at 37 °C for 24 h. Then, planktonic cells were removed, and the biofilms were stained with crystal violet solution (0.1%, *v*/*v*) for 1 h at 37 °C and then dissolved using glacial acetic acid (30%, *v*/*v*). The bacterial biofilms under the different treatments were quantized by measuring the absorbance at 570 nm using a BioTek SYNERGY H1 microplate reader (Agilnet, Santa Clara, CA, USA).

Additionally, to evaluate the eradication efficacy of mature biofilms by nordalbergin/amoxicillin alone or their combinations, MRSA USA300 was seeded into 24-well plates for 12 h, and then incubated with the indicated concentrations of nordalbergin/amoxicillin or their combinations for another 12 h at 37 °C. The bacterial biofilm was assessed as described above using crystal violet staining.

### 4.9. Scanning Electron Microscopy (SEM) Observation

Overnight MRSA USA300 was diluted and then incubated with nortriptyline/amoxicillin alone or their combination at 37 °C for 4 h. The bacteria were collected, washed, centrifuged, and resuspended in 2.5% glutaraldehyde. The samples were then observed for morphological changes using a Sigma 300 scanning electron microscopy (ZEISS, Oberkochen, Germany).

### 4.10. Live/Dead Bacteria Staining

Overnight cultured MRSA USA300 was incubated with nordalbergin, amoxicillin, or their combination for 4 h, and then washed with PBS and centrifuged. The collected bacteria were stained using the LIVE/DEAD BacLight Bacterial Viability Kit (Invitrogen, Carlsbad, CA, USA) according to the manufacturer’s protocol. Fluorescent images of stained bacteria were obtained using an inverted fluorescence microscope (Olympus, Tokyo, Japan). The green fluorescence-labeled bacteria were considered alive and the red fluorescence-labeled bacteria were considered dead.

### 4.11. Membrane Permeability Assay

Overnight cultured MRSA USA300 was incubated in LB Broth and then centrifuged in PBS and resuspended to obtain OD_600nm_ = 0.5. After treatment with various concentrations of nordalbergin, amoxicillin alone, or their combination for 1 h, the bacteria were incubated with 10 nM propidium iodide (PI) for 30 min. The fluorescence intensity was detected at Ex = 535 nm/Em = 615 nm using a BioTek SYNERGY H1 microplate reader (Agilnet, Santa Clara, CA, USA).

### 4.12. Membrane Depolarization Assay

MRSA USA300 (OD_600nm_ = 0.5) was incubated with 0.5 μM 3,3-Dipropylthiadicarbocyanine iodide [DiSC_3_(5)] for 30 min, and then treated with the indicated concentrations of nordalbergin/amoxicillin alone or their combination at 37 °C for 1 h. The dissipated membrane potentials of bacteria were measured with Ex = 622 nm/Em = 670 nm using a BioTek SYNERGY H1 microplate reader (Agilnet, Santa Clara, CA, USA).

### 4.13. NAD^+^/NADH Level Detection

MRSA USA300 in the logarithmic growth phase was adjusted to an OD_600nm_ of 0.8, centrifuged, resuspended in PBS, and treated with nordalbergin/amoxicillin alone, or their combination at 37 °C for 4 h. The bacterial cultures were collected, centrifuged, and resuspended. The NAD^+^/NADH ratio was determined using a NAD^+^/NADH Assay Kit (Beyotime, Shanghai, China).

### 4.14. ATP Determination

MRSA USA300 (OD_600nm_ = 0.5) was treated with nordalbergin, amoxicillin, or in combination for 4 h. After centrifugation at 12,000 rpm, the bacterial precipitates were lysed by ATP lysate and intra-bacterial ATP levels were measured using an Enhanced ATP Assay Kit (Beyotime, Shanghai, China) according to the kit instructions.

### 4.15. Animal Experiments

Female C57/BL6 mice aged 6–8 weeks were purchased from Liaoning Changsheng Technology Industrial Co., Ltd. (Liaoning, China) and housed with free access to food and water and acclimated for 5 days before experiments. MRSA USA300 (1 × 10^8^ CFU_S_) were administrated by the intranasal route and randomly divided into 6 mice/per group: MRSA-infection group, amoxicillin (40 mg/kg), nordalbergin (40 mg/kg or 80 mg/kg), and their combinations. All the drugs were dissolved using 0.5% carboxymethylcellulose sodium and administrated via oral gavage at 12 h intervals. The mice were euthanized at 24 h post-infection, and the lung tissues were collected and homogenized in ice-cold PBS for colony colonization and enzyme-linked immunosorbent assay (ELISA). The contents of cytokines TNF-α and IL-10 in lung tissue homogenate were measured by ELISA kits (BioLegend, Beijing, China) according to the manufacturer’s instructions. The lung tissues were preserved in 4% neutral buffered formalin for histopathological studies.

### 4.16. Statistical Analysis

Data were expressed as the means ± SD and examined for their statistically significant differences with the analysis of variance (ANOVA) and Student’s *t*-test. The *p*-values of less than 0.05 were statistically significant. The calculations and graphs were performed using GraphPad Prism 9.0 software (GraphPad Software, San Diego, CA, USA).

## 5. Conclusions

The current study validated the synergistic activity of nordalbergin and amoxicillin against MRSA in vivo and in vitro. In mechanisms, nordalbergin exerted synergistic effects against MRSA with amoxicillin through destroying MRSA biofilms and inhibiting *β*-lactamase performance. Our findings provided a novel *β*-lactam antibiotics potentiator for fighting against drug-resistant bacterial infections.

## Data Availability

The data are available from the corresponding author upon reasonable request.
